# Prediction of readmission in patients with acute exacerbation of chronic obstructive pulmonary disease within one year after treatment and discharge

**DOI:** 10.1186/s12890-021-01692-3

**Published:** 2021-10-15

**Authors:** Lili Chen, Shiping Chen

**Affiliations:** Department of Respiratory and Critical Care Medicine, The Second Affiliated Hospital of Nanjing Medical University, Jiangjiayuan 121#, Gulou District, Nanjing, 210000 Jiangsu China

**Keywords:** Acute exacerbation of chronic obstructive pulmonary disease, Readmission, Logistic model, XGBoost model

## Abstract

**Background:**

To investigate the risk factors and construct a logistic model and an extreme gradient boosting (XGBoost) model to compare the predictive performances for readmission in acute exacerbation of chronic obstructive pulmonary disease (AECOPD) patients within one year.

**Methods:**

In total, 636 patients with AECOPD were recruited and divided into readmission group (n = 449) and non-readmission group (n = 187). Backward stepwise regression method was used to analyze the risk factors for readmission. Data were divided into training set and testing set at a ratio of 7:3. Variables with statistical significance were included in the logistic model and variables with *P* < 0.1 were included in the XGBoost model, and receiver operator characteristic (ROC) curves were plotted.

**Results:**

Patients with acute exacerbations within the previous 1 year [odds ratio (OR) = 4.086, 95% confidence interval (CI) 2.723–6.133, *P* < 0.001), long-acting β agonist (LABA) application (OR = 4.550, 95% CI 1.587–13.042, *P* = 0.005), inhaled corticosteroids (ICS) application (OR = 0.227, 95% CI 0.076–0.672, *P* = 0.007), glutamic-pyruvic transaminase (ALT) level (OR = 0.985, 95% CI 0.971–0.999, *P* = 0.042), and total CAT score (OR = 1.091, 95% CI 1.048–1.136, *P* < 0.001) were associated with the risk of readmission. The AUC value of the logistic model was 0.743 (95% CI 0.692–0.795) in the training set and 0.699 (95% CI 0.617–0.780) in the testing set. The AUC value of XGBoost model was 0.814 (95% CI 0.812–0.815) in the training set and 0.722 (95% CI 0.720–0.725) in the testing set.

**Conclusions:**

The XGBoost model showed a better predictive value in predicting the risk of readmission within one year in the AECOPD patients than the logistic regression model. The findings of our study might help identify patients with a high risk of readmission within one year and provide timely treatment to prevent the reoccurrence of AECOPD.

**Supplementary Information:**

The online version contains supplementary material available at 10.1186/s12890-021-01692-3.

## Background

Chronic obstructive pulmonary disease (COPD) is a disease associated with chronic airway inflammation, characterized by persistent respiratory symptoms and airflow limitations [1]. COPD is the fourth major cause of death and may rise to the third leading cause of death by 2030 according to the prediction of the World Health Organization [2]. A national cross-sectional study in 2018 investigated the lung health status of adults > 20 years old in 10 provinces of China, and showed that the prevalence of COPD in adults > 20 years old was 8.6%, in adults > 40 years old was as high as 13.7%, causing a significant disease burden [3]. Acute exacerbation of COPD (AECOPD) refers to the aggravation of respiratory symptoms in patients, which is the main reason for hospitalization and medical expenditure of COPD patients [1, 4]. Approximately 63% of COPD patients have at least one readmission due to exacerbation within 1 year after hospitalization [5, 6]. AECOPD accelerate the progress of the disease, reduce the quality of life of patients and increase the risk of death [7]. Early identification of patients with high risk of AECOPD and readmission and timely interventions to reduce the incidence of AECOPD and readmission are of great clinical significance for improving the prognosis of COPD patients and delaying the progression of the disease.

Previously, the risk factors associated with AECOPD and readmission in patients were explored by several studies, which revealed that gender, hospital stay, medical aid care, duration of systemic steroid use were factors leading to the AECOPD and readmission [8]. Factors including age, tobacco use, diabetes mellitus, infections, obesity, and frequency of hospital visit were also reported to influencing the occurrence of AECOPD and readmission [9]. Currently, there was no international universal prediction model for predicting the readmission of AECOPD patients within one year after discharge. Prediction models of readmission in patients with AECOPD were established based on the data of USA or UK people and some of them were focused on predicting the risk of readmission of AECOPD patients within 30 days based on social factors or LACE index (length of stay, acuity of admission, co-morbidities, and emergency department visits within the last 6 months) [10, 11]. Additionally, the prediction models for readmission of AECOPD patients within 90 days were also established based on PEARL (previous admissions, eMRCD score, Age, Right-sided heart failure and Left-sided heart failure) or COPD-2-HOME score (CAT score, hyperinflation, obstruction, prior admission, eosinophilia) [12, 13]. A prediction model of readmission of AECOPD patients within 90 days considered the importance of multimorbidity, frailty and poor socioeconomic status in patients [14]. Njoku et al. [15] indicated that the prevalence of COPD-related readmission was about 2.6–82.2% within 30 days, 11.8–44.8% at 31–90 days, 17.9–63.0% at 6 months, and 25.0–87.0% at 12-month post-discharge [15], which suggested that the importance of not only predicted the readmission of AECOPD patients within 30 days or 90 days, but also one year. At present, a prediction model for one-year readmission of COPD patients was established but it had a low area under the curve (AUC) value and lacked validation of the results [16]. There was no prediction model for predicting the readmission of AECOPD patients within one year after discharge based on the data from Chinese population. To establish a prediction model for predicting the readmission of AECOPD patients within one year after discharge in China is of great value.

Gradient boosting machine (GBM) is a kind of machine learning algorithm helping assemble the weak learners into a strong learner. GBM increases the performance of the prediction model during the gradient descent process. The extreme gradient boosting (XGBoost) model is an extension of GBM, which combines several learning algorithms to achieve a better predictive performance than any of the constituent learning algorithms alone [17]. XGBoost applies a second-order Taylor expansion to the loss function and simultaneously implements the first derivative and the second derivative. Additionally, a regularization term is supplemented in the objective function to increase the generalizability of a single tree and decrease the complexity of the objective function [18]. XGBoost model is widely used for disease diagnosis and prediction due to its fast speed, excellent classification effect.

In our study, we collected the data of 650 patients with AECOPD from the Second Affiliated Hospital of Nanjing Medical University from Jan. 2016 to Dec. 2019 to investigate the risk factors and construct XGBoost model and logistic regression model to compare the predictive performance for readmission in AECOPD patients within one year after treatment and discharge.

## Methods

### Study population

**Fig. 1:**
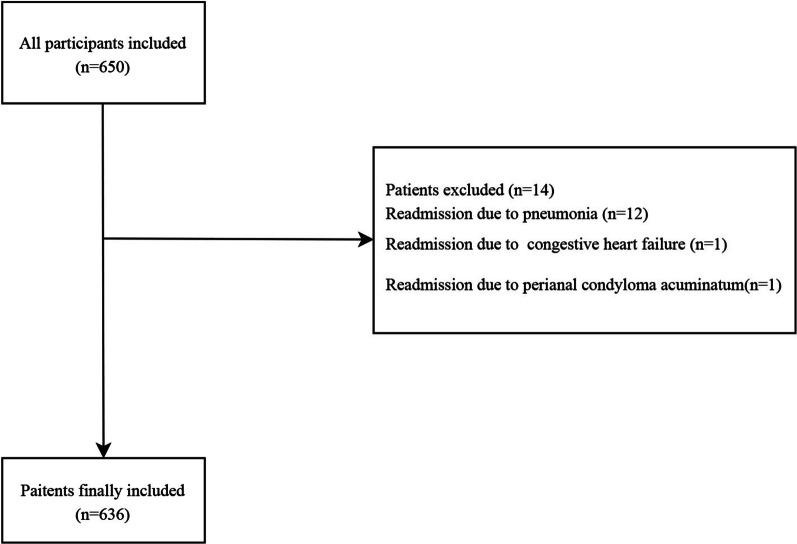
The screen process of the participants in this study

In the current study, 650 patients with AECOPD were recruited from the Second Affiliated Hospital of Nanjing Medical University between Jan. 2016 and Dec. 2019. The data of the patients were retrospectively extracted from a broad coding records search and review of COPD assessments routinely completed by clinicians or nurses. After excluding 12 patients who readmitted into hospitals because of pneumonia, 1 patient who readmitted into hospitals due to congestive heart failure and 1 patient who readmitted into hospitals due to perianal condyloma acuminatum, 636 participants were finally included. A hospitalization for AECOPD was identified through International Classification of Diseases-10 codes (J44.1) [19]. Readmission one year after discharge means within one year from their first day of discharge to readmission day [20]. All subjects were divided into readmission group (n = 187) and non-readmission group (n = 449). This study got the approval from the Ethics Committee of from the Second Affiliated Hospital of Nanjing Medical University, the approval number was (No. [2021]-KY-091-01). The screen process of the participants was shown in Fig. 1.

### Data collection

The data of participants were collected to analyze the risk factors of readmission within one year. The gender, age (years), body mass index (BMI, kg/m^2^), acute exacerbation in previous 1 year, smoking status, daily amount of smoking, duration of smoking, number of years of smoking packets, the application of long-acting muscarinic antagonist (LAMA), long-acting β agonist (LABA), short-acting muscarinic antagonist (SAMA), short-acting β agonist (SABA), phosphodiesterase 4 inhibitor (PDE4I), inhaled corticosteroids (ICS), history of congestive heart-failure, diabetes, hypertension, atrial fibrillation and combined with other disease were collected during the recruit time. The systolic blood pressure (mmHg), diastolic blood pressure (mmHg), respiratory rate (time/minute), temperature (℃), heart rate (time/minute), hemoglobin (Hb, g/L), red blood cells (RBC, 10^12^/L), white blood cells (WBC, 10^9^/L), platelets (PLT, 10^9^/L), neutrophil count (NEUT, 10^9^/L), percentage of neutrophils (%), lymphocyte count (LYM, 10^9^/L), percentage of lymphocytes (%), monocytes count (MONO, 10^9^/L), percentage of monocytes (%), eosinophil count (EOS, 10^9^/L), percentage of eosinophils (%), red blood cell distribution width (RDW), glutamic-pyruvic transaminase (ALT, μ/L), glutamic oxalacetic transaminase (AST, μ/L), total bilirubin (TBIL, μmol/L), albumin (ALB; g/L), blood urea nitrogen (BUN; μmol/L), creatinine (Cr, μmol/L), uric acid (μmol/L), hypersensitive C-reactive protein (mg/dL), modified Medical Research Council (mMRC) grade, and total COPD assessment test (CAT) score were collected at admission. The data on forced expiratory volume in 1 s (FEV_1,_ mL), forced vital capacity (FVC, mL), FEV_1_/FVC, FEV1 in predicted value FEV1, the use of systemic glucocorticoid, antibacterial agents, oxygen therapy, and mechanical ventilation were collected during the treatment. During the 12 months ± 30 days’ follow-up, if readmission occurred, the readmission frequency and reasons were recorded.

### Definitions of the variables

Congestive heart failure referred to the symptoms and/or signs of present heart failure, and left ventricular ejection fraction (LVEF) < 40%; or LVEF ≥ 40% with elevated brain natriuretic peptide and meeting at least one of the following requirements: (1) left ventricular hypertrophy and/or left atrial enlargement; (2) abnormal diastolic function.

Diabetes was defined as patients with blood glucose level ≥ 11.1 mmol/L at any time after meal or fasting blood glucose level ≥ 7.0 mmol/L, or having blood glucose level ≥ 11.1 mmol/L in 2-h glucose tolerance test or glycosylated hemoglobin ≥ 6.3%.

Hypertension was defined as systolic blood pressure ≥ 140 mmHg and/or diastolic blood pressure ≥ 90 mmHg when blood pressure was measured three times on different days without using antihypertensive drugs; For patients with a history of hypertension and currently taking antihypertensive drugs, they were diagnosed with hypertension although the blood pressure was lower than 140/90 mmHg.

Smoking status: including never smoking, former smoking and current smoking. Non-smoking was defined as less than 100 cigarettes in a lifetime, former smoking referred to more than 1 year of smoking cessation, and number of years of smoking packets = number of smoking packets per day (20 cigarettes are counted as 1 packet) × smoking years [21].

### XGBoost model

XGBoost model is an ensemble learning algorithm based on the gradient-boosted tree algorithm. XGBoost model processes sparse data via a sparsity-aware learning algorithm and weights quantile sketch to approximate tree learning [22].

### Statistical analysis

All statistical tests were conducted by two-sided test. The measurement data of normal distribution were described by Mean ± standard deviation (Mean ± SD), the independent sample t test was applied for comparisons between groups. The non-normal distributed data were expressed by median and quaternary spacing [M (Q1, Q3)], and differences between groups were compared by the Mann–Whitney U rank sum test. The enumeration data were shown as n (%). Chi-square test or Fisher’s exact probability method was used for comparison between groups. Random forest filling method was applied for filling in missing values with 100 trees via the missForest package in R© Version 3.5.1 (R Foundation for Statistical Computing, Vienna, Austria) [23]. Sensitivity analysis was performed before and after interpolation. To explore the risk factors for readmission in AECOPD patients within one year, the differences were firstly analyzed between groups, and the variables with statistical significance were included in the multivariate logistic model. Backward stepwise regression method was used to analyze the risk factors for readmission. For the establishment of prediction models, 70% of the samples were involved as the training set for construction of the models, and 30% of the samples were used as the testing set to test the diagnostic efficiency of the models [24, 25], and the equilibria analysis was conducted between the training set and the testing set. Variables with *P* < 0.1 were included in the logistic model and the extreme gradient boosting (XGBoost) model, and the parameters were adjusted. After establishing the models, the area under the curve (AUC) value, kolmogorov–smirnov (KS), sensitivity, specificity and accuracy were used to evaluate the performance of models. The receiver operator characteristic (ROC) curves were plotted. SAS 9.4 and R 3.6 were employed for data analysis in our study, and *P* < 0.05 referred to be statistical significant.

## Results

### The manipulation of missing data

Variables with a missing value ratio of more than 25% were removed (most of them were data related to discharge including partial arterial oxygen pressure, partial pressure of carbon dioxide in artery, arterial oxygenation, pH, and medications at discharge), and random forest filling method was used to fill in missing values for selected data. Sensitivity analysis before and after interpolation was shown in Table 1. There was no bias after interpolation of the missing data.

**Table 1:** Sensitivity analysis of the data before and after interpolation

Variable	Before interpolation (n = 636)	After interpolation (n = 636)	Statistical magnitude	*P*
Gender, n (%)			χ^2^ = 0.000	1.000
Male	425 (66.82)	425 (66.82)		
Female	211 (33.18)	211 (33.18)		
Age, mean ± SD	70.82 ± 9.88	70.82 ± 9.88	t = 0.000	1.000
Height, mean ± SD	1.67 ± 0.07	1.67 ± 0.07	t = 0.000	1.000
Weight, mean ± SD	65.50 ± 8.44	65.50 ± 8.44	t = 0.000	1.000
Acute exacerbation in previous 1 year, n (%)			χ^2^ = 0.000	1.000
No	430 (67.61)	430 (67.61)		
Yes	206 (32.39)	206 (32.39)		
Smoking status, n(%)			χ^2^ = 0.000	1.000
Never smoked	321 (50.47)	321 (50.47)		
Smoked before	93 (14.62)	93 (14.62)		
Smoker at present	222 (34.91)	222 (34.91)		
Daily amount of smoking, M (Q_1_, Q_3_)	0.00 (0.00, 15.00)	0.00 (0.00, 15.00)	Z = 0.000	1.000
Duration of smoking, M (Q_1_, Q_3_)	0.00 (0.00, 30.00)	0.00 (0.00, 30.00)	Z = 0.000	1.000
LAMA, n (%)			χ^2^ = 0.000	1.000
No	479 (75.31)	479 (75.31)		
Yes	157 (24.69)	157 (24.69)		
SAMA, n (%)			χ^2^ = 0.000	1.000
No	634 (99.69)	634 (99.69)		
Yes	2 (0.31)	2 (0.31)		
LABA, n (%)			χ^2^ = 0.000	1.000
No	413 (64.94)	413 (64.94)		
Yes	223 (35.06)	223 (35.06)		
SABA, n (%)			χ^2^ = 0.000	1.000
No	555 (87.26)	555 (87.26)		
Yes	81 (12.74)	81 (12.74)		
PDE4I, n (%)			χ^2^ = 0.000	1.000
No	617 (97.01)	617 (97.01)		
Yes	19 (2.99)	19 (2.99)		
ICS, n (%)			χ^2^ = 0.000	1.000
No	422 (66.35)	422 (66.35)		
Yes	214 (33.65)	214 (33.65)		
Congestive heart-failure, n (%)			χ^2^ = 0.000	1.000
No	572 (89.94)	572 (89.94)		
Yes	64 (10.06)	64 (10.06)		
Diabetes, n (%)			χ^2^ = 0.000	1.000
No	498 (78.30)	498 (78.30)		
Yes	138 (21.70)	138 (21.70)		
Hypertension, n (%)			χ^2^ = 0.000	1.000
No	280 (44.03)	280 (44.03)		
Yes	356 (55.97)	356 (55.97)		
Atrial fibrillation, n (%)			χ^2^ = 0.000	1.000
No	596 (93.71)	596 (93.71)		
Yes	40 (6.29)	40 (6.29)		
Combined with other diseases, n (%)			χ^2^ = 0.000	1.000
No	432 (67.92)	432 (67.92)		
Yes	204 (32.08)	204 (32.08)		
Systolic blood pressure, Mean ± SD	133.46 ± 17.34	133.46 ± 17.34	t = 0.000	1.000
Diastolic blood pressure, Mean ± SD	78.73 ± 10.82	78.73 ± 10.82	t = 0.000	1.000
Respiratory rate, Mean ± SD	19.28 ± 2.53	19.28 ± 2.53	t = 0.000	1.000
Temperature, mean ± SD	36.86 ± 0.56	36.86 ± 0.56	t = 0.000	1.000
Heart rate, mean ± SD	81.65 ± 11.35	81.65 ± 11.35	t = 0.000	1.000
Hb, mean ± SD	132.50 ± 17.34	132.51 ± 17.26	t = − 0.002	0.998
RBC, mean ± SD	4.33 ± 0.55	4.33 ± 0.55	t = 0.046	0.963
WBC, M (Q_1_, Q_3_)	6.89 (5.51, 8.97)	6.90 (5.53, 8.93)	Z = − 0.027	0.979
PLT, M (Q_1_, Q_3_)	188.00 (151.00, 235.75)	188.50 (151.75, 235.00)	Z = − 0.043	0.966
NEUT, M (Q_1_, Q_3_)	4.67 (3.52, 6.65)	4.68 (3.53, 6.65)	Z = − 0.044	0.965
Percentage of neutrophils, mean ± SD	70.51 ± 11.70	70.54 ± 11.65	t = − 0.052	0.959
LYM, M (Q_1_, Q_3_)	1.28 (0.90, 1.80)	1.29 (0.91, 1.80)	Z = − 0.079	0.937
Percentage of lymphocytes, M (Q_1_, Q_3_)	19.40 (12.40, 27.30)	19.43 (12.47, 27.13)	Z = 0.019	0.985
MONO, M (Q_1_, Q_3_)	0.44 (0.33, 0.60)	0.44 (0.33, 0.60)	Z = − 0.066	0.947
Percentage of monocytes, M (Q_1_, Q_3_)	6.30 (4.90, 8.10)	6.30 (4.90, 8.00)	Z = 0.045	0.964
EOS, M (Q_1_, Q_3_)	0.10 (0.03, 0.19)	0.10 (0.03, 0.19)	Z = − 0.036	0.971
Percentage of eosinophils, M(Q_1_, Q_3_)	1.50 (0.40, 2.90)	1.46 (0.40, 2.90)	Z = 0.018	0.986
RDW, mean ± SD	13.20 ± 1.07	13.20 ± 1.05	t = − 0.020	0.984
ALT, M (Q_1_, Q_3_)	15.45 (10.93, 23.00)	15.50 (11.00, 23.00)	Z = − 0.027	0.978
AST, M (Q_1_, Q_3_)	18.00 (14.00, 23.17)	18.00 (14.00, 23.10)	Z = − 0.026	0.979
TBIL, M (Q_1_, Q_3_)	9.30 (6.65, 13.35)	9.35 (6.70, 13.30)	Z = − 0.013	0.989
ALB, mean ± SD	39.88 ± 4.68	39.89 ± 4.67	t = − 0.035	0.972
BUN, M (Q_1_, Q_3_)	5.44 (4.44, 6.68)	5.44 (4.45, 6.68)	Z = − 0.014	0.989
Cr, M (Q_1_, Q_3_)	76.00 (64.10, 93.90)	76.20 (64.40, 93.75)	Z = − 0.037	0.971
Uric acid, M (Q_1_, Q_3_)	288.00 (226.00, 352.00)	288.50 (226.00, 352.00)	Z = − 0.009	0.993
Hypersensitive C-reactive protein, M (Q_1_, Q_3_)	8.30 (2.00, 38.00)	8.40 (2.00, 36.67)	Z = − 0.655	0.512
mMRC grade, n (%)			Z = 0.000	1.000
0	1 (0.16)	1 (0.16)		
1	138 (21.70)	138 (21.70)		
2	279 (43.87)	279 (43.87)		
3	189 (29.72)	189 (29.72)		
4	29 (4.56)	29 (4.56)		
FEV_1_, M (Q_1_, Q_3_)	1.60 (1.10, 2.21)	1.60 (1.10, 2.21)	Z = 0.000	1.000
FVC, M (Q_1_, Q_3_)	2.26 (1.66, 3.64)	2.26 (1.66, 3.64)	Z = 0.000	1.000
FEV_1_/FVC, mean ± SD	67.25 ± 13.27	67.25 ± 13.27	t = 0.000	1.000
FEV_1_ in predicted value FEV_1_, mean ± SD	55.33 ± 17.82	55.33 ± 17.82	t = 0.000	1.000
Use of systemic glucocorticoid, n (%)			χ^2^ = 0.000	1.000
No	472 (74.21)	472 (74.21)		
Yes	164 (25.79)	164 (25.79)		
Antibacterial agents, n (%)			χ^2^ = 0.000	1.000
No	13 (2.04)	13 (2.04)		
Yes	623 (97.96)	623 (97.96)		
Oxygen therapy, n (%)			χ^2^ = 0.000	1.000
No	126 (19.81)	126 (19.81)		
Yes	510 (80.19)	510 (80.19)		
Mechanical ventilation, n (%)			χ^2^ = 0.000	1.000
No	628 (98.74)	628 (98.74)		
Noninvasive ventilation	8 (1.26)	8 (1.26)		
Group, n (%)			χ^2^ = 0.000	1.000
Non-readmission group	449 (70.60)	449 (70.60)		
Readmission group	187 (29.40)	187 (29.40)		
BMI, mean ± SD	23.48 ± 2.95	23.48 ± 2.95	t = 0.000	1.000
CAT score, mean ± SD	20.28 ± 5.34	20.28 ± 5.34	t = 0.000	1.000

*LAMA* long-acting muscarinic antagonist, *LABA* long-acting β agonist, *SAMA* short-acting muscarinic antagonist, *SABA* short-acting β agonist, *PDE4I* phosphodiesterase 4 inhibitor, *ICS* inhaled corticosteroids, *Hb* hemoglobin, *RBC* red blood cells, *WBC* white blood cells, *PLT* platelets, *NEUT* neutrophil count, *LYM* lymphocyte count, *MONO* monocytes count, *EOS* eosinophil count, *RDW* red blood cell distribution width, *ALT* glutamic-pyruvic transaminase, *AST* glutamic oxalacetic transaminase, *TBIL* total bilirubin, *ALB* albumin, *BUN* blood urea nitrogen, *Cr* creatinine, *mMRC* modified Medical Research Council, *CAT* COPD assessment test, *FEV*_*1*_ forced expiratory volume in 1 s, *FVC* forced vital capacity

### Comparisons of baseline data between readmission group and non-readmission group

As exhibited in Table 2, the age (72.21 years vs. 70.24 years, t =  − 2.295, *P* = 0.022), MONO counts (0.48 10^9^/L vs. 0.43 10^9^/L, Z = 2.438, *P* = 0.015), mMRC grade (t = 5.963, *P* < 0.001), total CAT score (22,04 vs. 19.55, t =  − 5.475, *P* < 0.001), the proportions of patients with acute exacerbation in previous 1 year (54.01% vs. 23.39%, χ^2^ = 56.542, *P* < 0.001), patients using LAMA (32.09% vs. 21.60%, χ^2^ = 7.802, *P* = 0.005), LABA (47.06% vs. 30.07%, χ^2^ = 16.741, *P* < 0.001), ICS (43.32% vs. 29.62%, χ^2^ = 11.089, *P* < 0.001), and patients receiving systemic glucocorticoids (32.62% vs. 22.94%, χ^2^ = 6.465, *P* = 0.011), oxygen therapy (85.03% vs. 78.17%, χ^2^ = 3.903, *P* = 0.048) and mechanical ventilation (2.67% vs. 0.67%, χ^2^ = 4.276, *P* = 0.039) in the readmission group were higher than in the non-readmission group. The percentage of lymphocytes (17.60 vs. 19.80, Z =  − 2.031, *P* = 0.042), ALT (13.30 μ/L vs. 16.22 μ/L, Z =  − 3.176, *P* = 0.002), AST (16.50 μ/L vs. 19.00 μ/L, Z =  − 2.896, *P* = 0.004), FEV_1_/FVC (63.36 vs. 68.87, t = 4.845, *P* < 0.001) and the predicted value of FEV1 (52.60 vs. 58.40, Z =  − 3.076, *P* = 0.002) in the readmission group were lower than in the non-readmission group.

**Table 2:** Comparisons of characteristics of patients between readmission group and non-readmission group

Variable	Description (n = 636)	Non-readmission group (n = 449)	Readmission group (n = 187)	Statistical magnitude	*P*
Gender, n (%)				χ^2^ = 2.208	0.137
Male	425 (66.82)	292 (65.03)	133 (71.12)		
Female	211 (33.18)	157 (34.97)	54 (28.88)		
Age, mean ± SD	70.82 ± 9.88	70.24 ± 9.73	72.21 ± 10.09	t = − 2.295	0.022
BMI, mean ± SD	23.48 ± 2.95	23.50 ± 2.93	23.41 ± 2.99	t = 0.375	0.708
Acute exacerbation in previous 1 year, n (%)				χ^2^ = 56.542	< 0.001
No	430 (67.61)	344 (76.61)	86 (45.99)		
Yes	206 (32.39)	105 (23.39)	101 (54.01)		
Smoking status, n(%)				Z = − 1.393	0.164
Never smoked	321 (50.47)	220 (49.00)	101 (54.01)		
Smoked before	93 (14.62)	64 (14.25)	29 (15.51)		
Smoker at present	222 (34.91)	165 (36.75)	57 (30.48)		
Daily amount of smoking, M (Q_1_, Q_3_)	0.00 (0.00, 15.00)	0.00 (0.00, 15.00)	0.00 (0.00, 10.00)	Z = − 1.499	0.134
Duration of smoking, M (Q_1_, Q_3_)	0.00 (0.00, 30.00)	0.00 (0.00, 30.00)	0.00 (0.00, 30.00)	Z = − 1.496	0.135
Number of years of smoking packets, M (Q_1_, Q_3_)	0.00 (0.00, 21.00)	0.00 (0.00, 25.00)	0.00 (0.00, 20.00)	Z = − 1.597	0.110
LAMA, n(%)				χ^2^ = 7.802	0.005
No	479 (75.31)	352 (78.40)	127 (67.91)		
Yes	157 (24.69)	97 (21.60)	60 (32.09)		
SAMA, n(%)				χ^2^ = 0.410	0.522
No	634 (99.69)	448 (99.78)	186 (99.47)		
Yes	2 (0.31)	1 (0.22)	1 (0.53)		
LABA, n(%)				χ^2^ = 16.741	< 0.001
No	413 (64.94)	314 (69.93)	99 (52.94)		
Yes	223 (35.06)	135 (30.07)	88 (47.06)		
SABA, n(%)				χ^2^ = 0.993	0.319
No	555 (87.26)	388 (86.41)	167 (89.30)		
Yes	81 (12.74)	61 (13.59)	20 (10.70)		
PDE4I, n(%)				χ^2^ = 0.045	0.833
No	617 (97.01)	436 (97.10)	181 (96.79)		
Yes	19 (2.99)	13 (2.90)	6 (3.21)		
ICS, n(%)				χ^2^ = 11.089	< 0.001
No	422 (66.35)	316 (70.38)	106 (56.68)		
Yes	214 (33.65)	133 (29.62)	81 (43.32)		
Congestive heart-failure, n (%)				χ^2^ = 0.399	0.528
No	572 (89.94)	406 (90.42)	166 (88.77)		
Yes	64 (10.06)	43 (9.58)	21 (11.23)		
Diabetes, n (%)				χ^2^ = 0.008	0.929
No	498 (78.30)	352 (78.40)	146 (78.07)		
Yes	138 (21.70)	97 (21.60)	41 (21.93)		
Hypertension, n (%)				χ^2^ = 1.650	0.199
No	280 (44.03)	205 (45.66)	75 (40.11)		
Yes	356 (55.97)	244 (54.34)	112 (59.89)		
Atrial fibrillation, n (%)				χ^2^ = 0.197	0.657
No	596 (93.71)	422 (93.99)	174 (93.05)		
Yes	40 (6.29)	27 (6.01)	13 (6.95)		
Combined with other diseases, n (%)				χ^2^ = 0.034	0.855
No	432 (67.92)	304 (67.71)	128 (68.45)		
Yes	204 (32.08)	145 (32.29)	59 (31.55)		
Systolic blood pressure, mean ± SD	133.46 ± 17.34	133.57 ± 17.48	133.18 ± 17.01	t = 0.258	0.796
Diastolic blood pressure, mean ± SD	78.73 ± 10.82	78.87 ± 10.99	78.40 ± 10.40	t = 0.496	0.620
Respiratory rate, mean ± SD	19.28 ± 2.53	19.20 ± 2.52	19.47 ± 2.53	t = − 1.203	0.229
Temperature, mean ± SD	36.86 ± 0.56	36.87 ± 0.59	36.84 ± 0.46	t = 0.783	0.434
Heart rate, mean ± SD	81.65 ± 11.35	81.83 ± 11.85	81.22 ± 10.06	t = 0.616	0.538
Hb, mean ± SD	132.51 ± 17.26	132.80 ± 17.22	131.79 ± 17.32	t = 0.678	0.498
RBC, mean ± SD	4.33 ± 0.55	4.35 ± 0.56	4.28 ± 0.51	t = 1.386	0.166
WBC, M (Q_1_, Q_3_)	6.90 (5.53, 8.93)	6.89 (5.50, 8.83)	7.01 (5.71, 9.07)	Z = 0.922	0.356
PLT, M (Q_1_, Q_3_)	188.50 (151.75, 235.00)	190.00 (152.00, 234.00)	183.00 (149.50, 240.00)	Z = − 0.627	0.531
NEUT, M (Q_1_, Q_3_)	4.68 (3.53, 6.65)	4.64 (3.43, 6.65)	4.96 (3.82, 6.62)	Z = 1.334	0.182
Percentage of neutrophils, mean ± SD	70.54 ± 11.65	70.20 ± 11.65	71.37 ± 11.60	t = − 1.152	0.250
LYM, M (Q_1_, Q_3_)	1.29 (0.91, 1.80)	1.33 (0.92, 1.81)	1.23 (0.85, 1.67)	Z = − 1.389	0.165
Percentage of lymphocytes, M (Q_1_, Q_3_)	19.42 (12.47, 27.13)	19.80 (13.30, 27.80)	17.60 (11.25, 25.25)	Z = − 2.031	0.042
MONO, M (Q_1_, Q_3_)	0.44 (0.33, 0.60)	0.43 (0.33, 0.58)	0.48 (0.34, 0.67)	Z = 2.438	0.015
Percentage of monocytes, M (Q_1_, Q_3_)	6.30 (4.90, 8.00)	6.20 (4.90, 7.90)	6.50 (5.30, 8.30)	Z = 1.451	0.147
EOS, M (Q_1_, Q_3_)	0.10 (0.03, 0.19)	0.10 (0.03, 0.19)	0.10 (0.03, 0.19)	Z = 0.048	0.962
Percentage of eosinophils, M(Q_1_, Q_3_)	1.46 (0.40, 2.90)	1.47 (0.40, 2.90)	1.45 (0.40, 3.05)	Z = 0.007	0.995
RDW, Mean ± SD	13.20 ± 1.05	13.16 ± 1.03	13.29 ± 1.08	t = − 1.342	0.180
ALT, M (Q_1_, Q_3_)	15.50 (11.00, 23.00)	16.22 (11.10, 25.00)	13.30 (9.80, 19.95)	Z = − 3.176	0.002
AST, M (Q_1_, Q_3_)	18.00 (14.00, 23.10)	19.00 (14.70, 23.80)	16.50 (13.40, 21.30)	Z = − 2.896	0.004
TBIL, M (Q_1_, Q_3_)	9.35 (6.70, 13.30)	9.60 (6.90, 13.60)	8.70 (6.35, 12.30)	Z = − 1.947	0.052
ALB, Mean ± SD	39.89 ± 4.67	40.03 ± 4.73	39.55 ± 4.50	t = 1.198	0.231
BUN, M (Q_1_, Q_3_)	5.44 (4.45, 6.68)	5.45 (4.48, 6.66)	5.43 (4.39, 6.81)	Z = 0.053	0.958
Cr, M (Q_1_, Q_3_)	76.20 (64.40, 93.75)	75.40 (64.80, 90.90)	78.80 (63.70, 98.15)	Z = 1.099	0.272
Uric acid, M (Q_1_, Q_3_)	288.50 (226.00, 352.00)	292.00 (229.00, 351.00)	280.00 (224.50, 352.00)	Z = − 0.121	0.903
Hypersensitive C-reactive protein, M (Q_1_, Q_3_)	8.40 (2.00, 36.67)	7.20 (2.00, 38.00)	9.70 (2.15, 33.20)	Z = 0.429	0.668
mMRC grade, n (%)				Z = 5.763	< 0.001
0	1 (0.16)	0 (0.00)	1 (0.22)		
1	138 (21.70)	22 (11.76)	116 (25.84)		
2	279 (43.87)	73 (39.04)	206 (45.88)		
3	189 (29.72)	75 (40.11)	114 (25.39)		
4	29 (4.56)	17 (9.09)	12 (2.67)		
CAT score, Mean ± SD	20.28 ± 5.34	19.55 ± 5.02	22.04 ± 5.67	t = − 5.475	< 0.001
FEV_1_, M (Q_1_, Q_3_)	1.60 (1.10, 2.21)	1.62 (1.12, 2.21)	1.60 (1.05, 2.19)	Z = − 0.504	0.614
FVC, M (Q_1_, Q_3_)	2.26 (1.66, 3.64)	2.20 (1.65, 3.45)	2.34 (1.70, 3.83)	Z = 1.379	0.168
FEV1/FVC, Mean ± SD	67.25 ± 13.27	68.87 ± 13.48	63.36 ± 11.88	t = 4.845	< 0.001
FEV_1_ in predicted value FEV_1_, mean ± SD	56.81 (42.65, 67.80)	58.40 (43.70, 68.42)	52.60 (38.80, 65.70)	Z = − 3.076	0.002
Use of systemic glucocorticoid, n (%)				χ^2^ = 6.465	0.011
No	472 (74.21)	346 (77.06)	126 (67.38)		
Yes	164 (25.79)	103 (22.94)	61 (32.62)		
Antibacterial agents, n (%)				χ^2^ = 0.012	0.913
No	13 (2.04)	9 (2.00)	4 (2.14)		
Yes	623 (97.96)	440 (98.00)	183 (97.86)		
Oxygen therapy, n (%)				χ^2^ = 3.903	0.048
No	126 (19.81)	98 (21.83)	28 (14.97)		
Yes	510 (80.19)	351 (78.17)	159 (85.03)		
Mechanical ventilation, n (%)				χ^2^ = 4.276	0.039
No	628 (98.74)	446 (99.33)	182 (97.33)		
Noninvasive ventilation	8 (1.26)	3 (0.67)	5 (2.67)		

*LAMA* long-acting muscarinic antagonist, *LABA* long-acting β agonist, *SAMA* short-acting muscarinic antagonist, *SABA* short-acting β agonist, *PDE4I* phosphodiesterase 4 inhibitor, *ICS* inhaled corticosteroids, *Hb* hemoglobin, *RBC* red blood cells, *WBC* white blood cells, *PLT* platelets, *NEUT* neutrophil count, *LYM* lymphocyte count, *MONO* monocytes count, *EOS* eosinophil count, *RDW* red blood cell distribution width, *ALT* glutamic-pyruvic transaminase, *AST* glutamic oxalacetic transaminase, *TBIL* total bilirubin, *ALB* albumin, *BUN* blood urea nitrogen, *Cr* creatinine, *mMRC* modified Medical Research Council, *CAT* COPD assessment test, *FEV*_*1*_ forced expiratory volume in 1 s, *FVC* forced vital capacity

### Risk factors of readmission in patients with AECOPD within one year

Variables with statistical significance in comparisons of the baseline data were included in the multivariate logistic regression analysis. Backward stepwise regression method was adopted, and age and gender were adjusted. The results delineated that patient with acute exacerbations within the previous 1 year had a 4.086-fold higher risk of readmission than those without acute exacerbations within the previous 1 year (OR = 4.086, 95% CI 2.723–6.133, *P* < 0.001). Patients using LABA had a 4.550-fold higher risk of readmission than those not using LABA (OR = 4.550, 95% CI 1.587–13.042, *P* = 0.005). Patients receiving ICS decreased the risk of readmission by 0.773 times (OR = 0.227, 95% CI 0.076–0.672, *P* = 0.007) compared with those not receiving ICS. The risk of readmission was reduced by 0.015 times with the per unit increase of ALT (OR = 0.985, 95% CI 0.971–0.999, *P* = 0.042). Each 1-point increase in total CAT score was associated with a 1.091-fold increased risk of readmission (OR = 1.091, 95% CI 1.048–1.136, *P* < 0.001) (Table 3).

**Table 3:** Predictors analysis of readmission

Variable	*β*	*S.E*	Wald	*P*	OR	95% CI	
						Lower	Upper
Intercept	− 2.374	0.753	9.942	0.002			
Age	− 0.008	0.011	0.491	0.484	0.992	0.972	1.014
Gender							
Male	Ref						
Female	− 0.216	0.208	1.076	0.299	0.806	0.535	1.212
Acute exacerbation in previous 1 year							
No	Ref						
Yes	1.408	0.207	46.174	< 0.001	4.086	2.723	6.133
LABA							
No	Ref						
Yes	1.515	0.537	7.951	0.005	4.550	1.587	13.042
ICS							
No	Ref						
Yes	− 1.484	0.555	7.157	0.007	0.227	0.076	0.672
ALT	− 0.015	0.007	4.152	0.042	0.985	0.971	0.999
CAT Score	0.087	0.021	17.871	< 0.001	1.091	1.048	1.136

*LABA* long-acting β agonist, *ICS* inhaled corticosteroids, *ALT* glutamic-pyruvic transaminase, *CAT* COPD assessment test

### The equilibrium test of training set and testing set

All samples were randomly divided into the training set and the testing set (7:3). The results of equilibrium analysis after division showed that there was no statistical significance in the differences of variables between the training set and the testing set (Table 4).

**Table 4:** The equilibrium test of training set and testing set

Variable	Description (n = 636)	Training set (n = 445)	Testing set (n = 191)	Statistical magnitude	*P*
Gender, n (%)				χ^2^ = 0.383	0.536
Male	425 (66.82)	294 (66.07)	131 (68.59)		
Female	211 (33.18)	151 (33.93)	60 (31.41)		
Age, Mean ± SD	70.82 ± 9.88	70.62 ± 9.91	71.27 ± 9.80	t = − 0.757	0.449
BMI, Mean ± SD	23.48 ± 2.95	23.42 ± 3.01	23.61 ± 2.79	t = − 0.750	0.453
Acute exacerbation in previous 1 year, n (%)				χ^2^ = 1.740	0.187
No	430 (67.61)	308 (69.21)	122 (63.87)		
Yes	206 (32.39)	137 (30.79)	69 (36.13)		
Smoking status, n(%)				Z = 0.336	0.737
Never smoked	321 (50.47)	229 (51.46)	92 (48.17)		
Smoked before	93 (14.62)	59 (13.26)	34 (17.80)		
Smoker at present	222 (34.91)	157 (35.28)	65 (34.03)		
Daily amount of smoking, M (Q_1_, Q_3_)	0.00 (0.00, 15.00)	0.00 (0.00, 12.00)	0.00 (0.00, 15.00)	Z = − 0.135	0.893
Duration of smoking, M (Q_1_, Q_3_)	0.00 (0.00, 30.00)	0.00 (0.00, 30.00)	0.00 (0.00, 30.00)	Z = − 0.243	0.808
Number of years of smoking packets, M (Q_1_, Q_3_)	0.00 (0.00, 21.00)	0.00 (0.00, 25.00)	0.00 (0.00, 20.00)	Z = − 0.133	0.894
LAMA, n(%)				χ^2^ = 1.067	0.302
No	479 (75.31)	330 (74.16)	149 (78.01)		
Yes	157 (24.69)	115 (25.84)	42 (21.99)		
SAMA, n(%)				χ^2^ = 0.861	0.353
No	634 (99.69)	443 (99.55)	191 (100.00)		
Yes	2 (0.31)	2 (0.45)	0 (0.00)		
LABA, n(%)				χ^2^ = 0.035	0.852
No	413 (64.94)	290 (65.17)	123 (64.40)		
Yes	223 (35.06)	155 (34.83)	68 (35.60)		
SABA, n(%)				χ^2^ = 0.189	0.664
No	555 (87.26)	390 (87.64)	165 (86.39)		
Yes	81 (12.74)	55 (12.36)	26 (13.61)		
PDE4I, n(%)				χ^2^ = 0.129	0.720
No	617 (97.01)	431 (96.85)	186 (97.38)		
Yes	19 (2.99)	14 (3.15)	5 (2.62)		
ICS, n(%)				χ^2^ = 0.101	0.751
No	422 (66.35)	297 (66.74)	125 (65.45)		
Yes	214 (33.65)	148 (33.26)	66 (34.55)		
Congestive heart-failure, n (%)				χ^2^ = 0.004	0.950
No	572 (89.94)	400 (89.89)	172 (90.05)		
Yes	64 (10.06)	45 (10.11)	19 (9.95)		
Diabetes, n (%)				χ^2^ = 2.515	0.113
No	498 (78.30)	356 (80.00)	142 (74.35)		
Yes	138 (21.70)	89 (20.00)	49 (25.65)		
Hypertension, n (%)				χ^2^ = 0.507	0.476
No	280 (44.03)	200 (44.94)	80 (41.88)		
Yes	356 (55.97)	245 (55.06)	111 (58.12)		
Atrial fibrillation, n (%)				χ^2^ = 0.124	0.725
No	596 (93.71)	418 (93.93)	178 (93.19)		
Yes	40 (6.29)	27 (6.07)	13 (6.81)		
Combined with other diseases, n (%)				χ^2^ = 1.130	0.288
No	432 (67.92)	308 (69.21)	124 (64.92)		
Yes	204 (32.08)	137 (30.79)	67 (35.08)		
Systolic blood pressure, Mean ± SD	133.46 ± 17.34	132.66 ± 16.44	135.31 ± 19.16	t = − 1.665	0.097
Diastolic blood pressure, Mean ± SD	78.73 ± 10.82	78.21 ± 10.42	79.95 ± 11.62	t = − 1.859	0.063
Respiratory rate, Mean ± SD	19.28 ± 2.53	19.17 ± 2.41	19.52 ± 2.77	t = − 1.604	0.109
Temperature, Mean ± SD	36.86 ± 0.56	36.85 ± 0.54	36.90 ± 0.60	t = − 1.055	0.292
Heart rate, Mean ± SD	81.65 ± 11.35	81.40 ± 11.24	82.24 ± 11.60	t = − 0.860	0.390
Hb, Mean ± SD	132.51 ± 17.26	132.36 ± 17.41	132.85 ± 16.88	t = − 0.328	0.743
RBC, Mean ± SD	4.33 ± 0.55	4.32 ± 0.55	4.35 ± 0.54	t = − 0.654	0.514
WBC, M (Q_1_, Q_3_)	6.90 (5.53, 8.93)	6.85 (5.53, 8.68)	7.18 (5.50, 9.64)	Z = 1.141	0.254
PLT, M (Q_1_, Q_3_)	188.50 (151.75, 235.00)	189.00 (155.00, 234.00)	187.00 (144.50, 239.00)	Z = − 0.845	0.398
NEUT, M (Q_1_, Q_3_)	4.68 (3.53, 6.65)	4.54 (3.52, 6.41)	5.13 (3.54, 7.11)	Z = 1.663	0.096
Percentage of neutrophils, Mean ± SD	70.54 ± 11.65	69.96 ± 11.43	71.90 ± 12.03	t = − 1.926	0.055
LYM, M (Q_1_, Q_3_)	1.29 (0.91, 1.80)	1.28 (0.93, 1.80)	1.31 (0.85, 1.79)	Z = − 0.681	0.496
Percentage of lymphocytes, M (Q_1_, Q_3_)	19.42 (12.47, 27.13)	19.60 (12.80, 27.50)	18.90 (11.25, 26.30)	Z = − 1.593	0.111
MONO, M (Q_1_, Q_3_)	0.44 (0.33, 0.60)	0.44 (0.34, 0.60)	0.44 (0.33, 0.61)	Z = − 0.826	0.409
Percentage of monocytes, M (Q_1_, Q_3_)	6.30 (4.90, 8.00)	6.40 (5.00, 8.20)	6.10 (4.80, 7.80)	Z = − 1.907	0.057
EOS, M (Q_1_, Q_3_)	0.10 (0.03, 0.19)	0.10 (0.03, 0.19)	0.08 (0.03, 0.16)	Z = − 0.577	0.564
Percentage of eosinophils, M(Q_1_, Q_3_)	1.46 (0.40, 2.90)	1.50 (0.40, 3.00)	1.40 (0.40, 2.55)	Z = − 0.830	0.406
RDW, Mean ± SD	13.20 ± 1.05	13.20 ± 1.04	13.20 ± 1.06	t = 0.053	0.957
ALT, M (Q_1_, Q_3_)	15.50 (11.00, 23.00)	15.40 (11.00, 23.00)	15.60 (10.00, 23.35)	Z = − 0.244	0.807
AST, M (Q_1_, Q_3_)	18.00 (14.00, 23.10)	18.00 (14.30, 23.70)	17.30 (13.25, 22.20)	Z = − 1.658	0.097
TBIL, M (Q_1_, Q_3_)	9.35 (6.70, 13.30)	9.60 (6.80, 13.40)	9.00 (6.60, 12.45)	Z = − 0.492	0.623
ALB, Mean ± SD	39.89 ± 4.67	39.95 ± 4.78	39.74 ± 4.38	t = 0.521	0.602
BUN, M (Q_1_, Q_3_)	5.44 (4.45, 6.68)	5.47 (4.43, 6.77)	5.36 (4.51, 6.64)	Z = − 0.221	0.825
Cr, M (Q_1_, Q_3_)	76.20 (64.40, 93.75)	75.80 (64.00, 92.50)	78.70 (65.20, 93.95)	Z = 1.035	0.301
Uric acid, M (Q_1_, Q_3_)	288.50 (226.00, 352.00)	289.00 (232.00, 355.00)	287.00 (215.50, 347.00)	Z = − 0.829	0.407
Hypersensitive C-reactive protein, M (Q_1_, Q_3_)	8.40 (2.00, 36.67)	8.00 (2.20, 34.40)	9.30 (1.75, 43.15)	Z = 0.697	0.486
mMRC grade, n (%)				Z = 1.634	0.102
0	1 (0.16)	0 (0.00)	1 (0.52)		
1	138 (21.70)	103 (23.15)	35 (18.32)		
2	279 (43.87)	196 (44.04)	83 (43.46)		
3	189 (29.72)	131 (29.44)	58 (30.37)		
4	29 (4.56)	15 (3.37)	14 (7.33)		
CAT score, Mean ± SD	20.28 ± 5.34	20.16 ± 5.19	20.58 ± 5.67	t = − 0.917	0.360
FEV_1_, M (Q_1_, Q_3_)	1.60 (1.10, 2.21)	1.60 (1.09, 2.21)	1.64 (1.12, 2.20)	Z = 0.081	0.936
FVC, M (Q_1_, Q_3_)	2.26 (1.66, 3.64)	2.23 (1.65, 3.60)	2.30 (1.67, 3.67)	Z = 0.381	0.704
FEV1/FVC, Mean ± SD	67.25 ± 13.27	67.23 ± 13.17	67.30 ± 13.50	t = − 0.060	0.952
FEV_1_ in predicted value FEV_1_, Mean ± SD	55.33 ± 17.82	55.81 ± 17.73	54.19 ± 17.97	t = 1.048	0.295
Use of systemic glucocorticoid, n (%)				χ^2^ = 2.348	0.126
No	472 (74.21)	338 (75.96)	134 (70.16)		
Yes	164 (25.79)	107 (24.04)	57 (29.84)		
Antibacterial agents, n (%)				χ^2^ = 1.642	0.200
No	13 (2.04)	7 (1.57)	6 (3.14)		
Yes	623 (97.96)	438 (98.43)	185 (96.86)		
Oxygen therapy, n (%)				χ^2^ = 0.220	0.639
No	126 (19.81)	86 (19.33)	40 (20.94)		
Yes	510 (80.19)	359 (80.67)	151 (79.06)		
Mechanical ventilation, n (%)				χ^2^ = 0.098	0.755
No	628 (98.74)	439 (98.65)	189 (98.95)		
Noninvasive ventilation	8 (1.26)	6 (1.35)	2 (1.05)		
Group, n (%)				χ^2^ = 1.687	0.194
Non-readmission group	449 (70.60)	321 (72.13)	128 (67.02)		
Readmission group	187 (29.40)	124 (27.87)	63 (32.98)		

*LAMA* long-acting muscarinic antagonist, *LABA* long-acting β agonist, *SAMA* short-acting muscarinic antagonist, *SABA* short-acting β agonist, *PDE4I* phosphodiesterase 4 inhibitor, *ICS* inhaled corticosteroids, *Hb* hemoglobin, *RBC* red blood cells, *WBC* white blood cells, *PLT* platelets, *NEUT* neutrophil count, *LYM* lymphocyte count, *MONO* monocytes count, *EOS* eosinophil count, *RDW* red blood cell distribution width, *ALT* glutamic-pyruvic transaminase, *AST* glutamic oxalacetic transaminase, *TBIL* total bilirubin, *ALB* albumin, *BUN* blood urea nitrogen, *Cr* creatinine, *mMRC* modified Medical Research Council, *CAT* COPD assessment test, *FEV*_*1*_ forced expiratory volume in 1 s, *FVC* forced vital capacity

### Construction of logistic model and validation of the predicative value via the testing set

Variables with statistical differences were included in the logistic model. The stepwise backward method was used, and age and gender were included. The results were shown in Table 5. Patients with acute exacerbations within the previous 1 year had a 3.863 times higher risk of readmission than those without acute exacerbations within the previous 1 year (OR = 3.863, 95% CI 2.349–6.351, *P* < 0.001). LABA usage in patients increased the risk of readmission by 4.556 times (OR = 5.556, 95% CI 1.577–19.577, *P* = 0.008). ICS treatment decreased the risk of readmission by 0.753 than patients without ICS treatment (OR = 0.247, 95% CI 0.067–0.908, *P* = 0.035). Each 1-point increase in total CAT score was correlated with a 1.110-fold increased risk of readmission (OR = 1.110, 95% CI 1.055–1.168, *P* < 0.001).

**Table 5:** Construction of logistic model

Variable	*β*	*S.E*	Wald	*P*	OR	95% CI	
						Lower	Upper
Intercept	− 2.551	0.925	7.600	0.006			
Age	− 0.015	0.013	1.245	0.265	0.985	0.961	1.011
Gender							
Male	Ref						
Female	0.063	0.253	0.062	0.803	1.065	0.649	1.749
Acute exacerbation in previous 1 year							
No	Ref						
Yes	1.351	0.254	28.364	< 0.001	3.863	2.349	6.351
LABA							
No	Ref						
Yes	1.715	0.643	7.121	0.008	5.556	1.577	19.577
ICS							
No	Ref						
Yes	− 1.398	0.664	4.433	0.035	0.247	0.067	0.908
ALT	− 0.010	0.009	1.462	0.227	0.990	0.973	1.006
CAT Score	0.105	0.026	16.177	< 0.001	1.110	1.055	1.168

*LABA* long-acting β agonist, *ICS* inhaled corticosteroids, *ALT* glutamic-pyruvic transaminase, *CAT* COPD assessment test

**Fig. 2:**
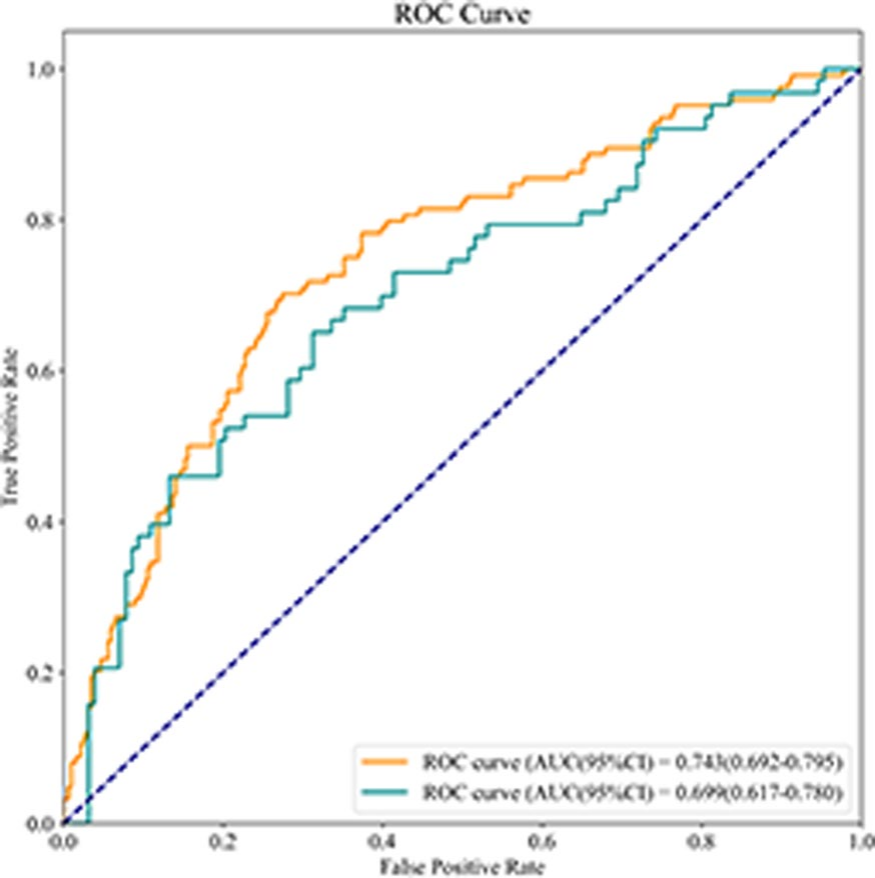
The ROC curve of showing the AUC value of the logistic model

The AUC value of the logistic model was 0.743 (95% CI 0.692–0.795) in the training set and 0.699 (95% CI 0.617–0.780) in the testing set (Fig. 2). The sensitivity was 0.702 (95% CI 0.621–0.782) in the training set and 0.667 (95% CI 0.550–0.783) in the testing set. The specificity was 0.726 (95% CI 0.677–0.775) in the training set and 0.664 (95% CI 0.582–0.746) in the testing set. The accuracy was 0.719 (95% CI 0.677–0.761) in the training set and 0.665 (95% CI 0.598–0.732) in the testing set. The cutoff point in the training set was 0.282 (Table 6).

**Table 6:** The predicative value of logistic model

Parameters	Data	
	Training set	Testing set
Sensitivity (95% CI)	0.702 (0.621–0.782)	0.667 (0.550–0.783)
Specificity (95% CI)	0.726 (0.677–0.775)	0.664 (0.582–0.746)
AUC (95% CI)	0.743 (0.692–0.795)	0.699 (0.617–0.780)
Accuracy (95% CI)	0.719 (0.677–0.761)	0.665 (0.598–0.732)
Cut off	0.282	–

*CI* confidence interval, *AUC* area under the curve

### Construction of XGBoost model and validation of the predicative value via the testing set

**Fig. 3:**
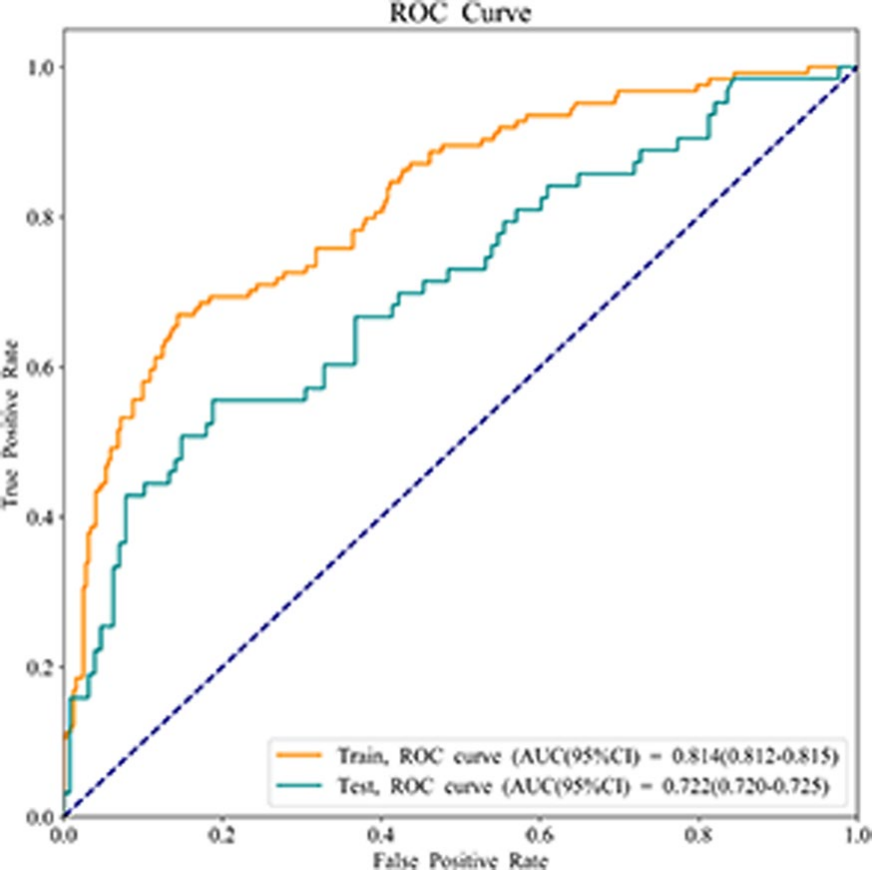
The weight method revealing the importance of variables in the XGBoost model

Variables with *P* < 0.1 were selected into the XGBoost model, and age and gender were also involved in. After GridSearchCV search tuning, the optimal parameters of the model were: tree depth: 2, number of trees: 50, learning rate: 0.01. The weight method was used to evaluate the importance of variables via the number of split nodes in the model tree. The results depicted that variables including acute exacerbation in previous 1 year, the CAT score, and SABA and LABA application were more important in the XGBoost model (Fig. 3).

**Fig. 4:**
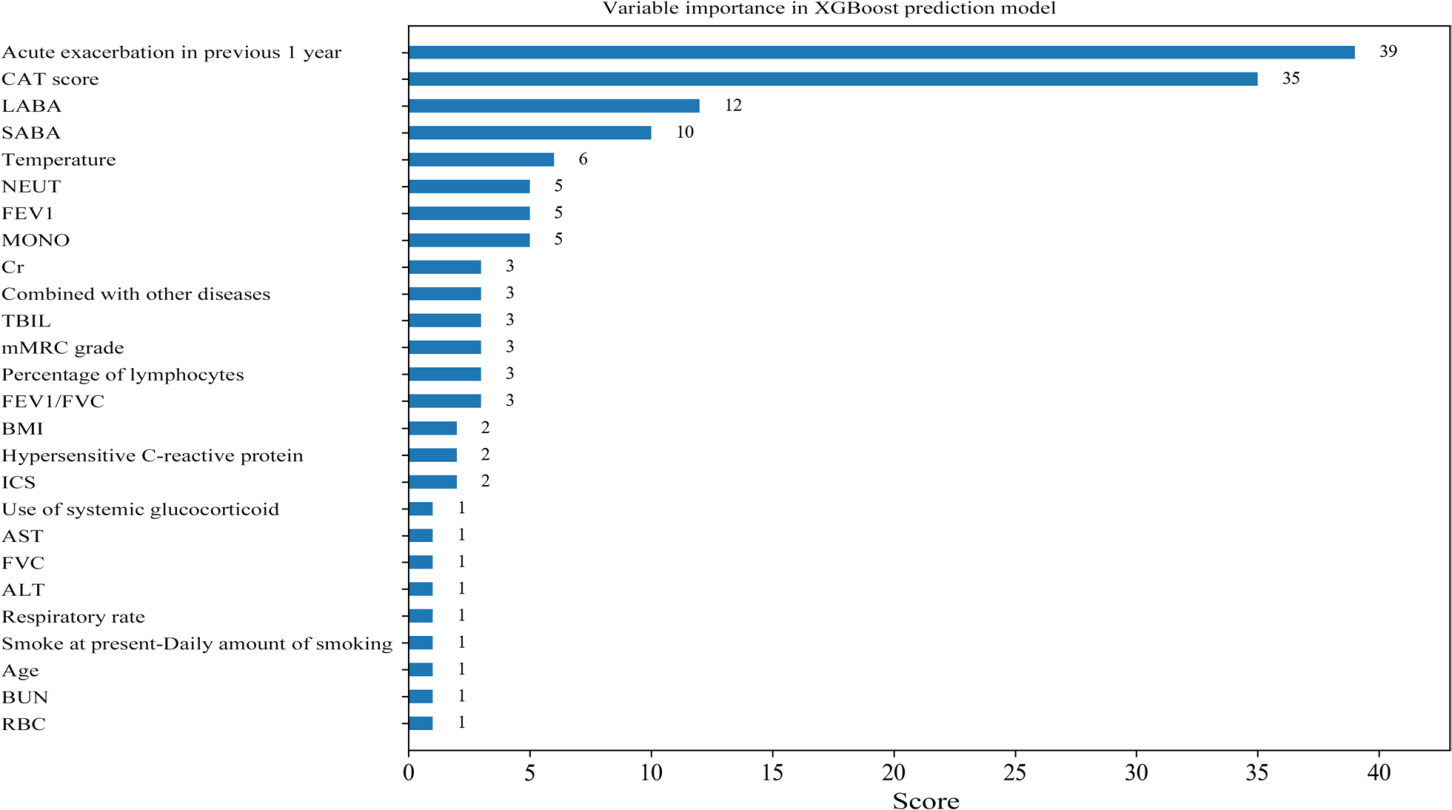
The ROC curve of showing the AUC value of the XGBoost model

As delineated in Table 7, the AUC value of XGBoost model was 0.814 (95% CI 0.812–0.815) in the training set and 0.722 (95% CI 0.720–0.725) in the testing set (Fig. 4). The sensitivity was 0.702 (95% CI 0.621–0.782) in the training set and 0.635 (95% CI 0.516–0.754) in the testing set. The specificity was 0.826 (95% CI 0.784–0.867) in the training set and 0.750 (95% CI 0.675–0.825) in the testing set. The accuracy was 0.791 (95% CI 0.753–0.829) in the training set and 0.712 (95% CI 0.648–0.776) in the testing set. The cut of point in the training set was 0.451.

**Table 7:** The predictive value of XGBoost model

Parameters	Data	
	Training set	Testing set
Sensitivity (95% CI)	0.702 (0.621–0.782)	0.635 (0.516–0.754)
Specificity (95% CI)	0.826 (0.784–0.867)	0.750 (0.675–0.825)
AUC (95% CI)	0.814 (0.812–0.815)	0.722 (0.720–0.725)
Accuracy (95%C I)	0.791 (0.753–0.829)	0.712 (0.648–0.776)

*CI* confidence interval, *AUC* area under the curve

### Comparisons of the predictive abilities of the two prediction models

The logistic model and XGBoost model were used to establish the prediction model, and the performance of the models were compared. The AUC value of the logistic model was good in the training set but poor in the testing set while the XGBoost model showed good predictive abilities in both the training set and the testing set.

## Discussion

This study collected the data of 650 patients with AECOPD and evaluated the risk factors for readmission within one year after treatment and discharge and constructed logistic model and XGBoost model to predict the risk of readmission within one year in AECOPD patients. The data revealed that acute exacerbation within the previous 1 year, LABA application, and the total CAT score were risk factors for readmission of AECOPD patients within one year while ICS application and higher ALT level were protective factors for readmission of AECOPD patients within one year. Additionally, we compared the predictive performances of logistic model and XGBoost model in predicting the risk of readmission within one year in AECOPD patients. The data delineated that the XGBoost model showed better predictive value.

Previously, the history of exacerbation was reported to an independent predictor for future exacerbations in patients [26]. A study of Bernabeu-Mora et al. indicated that the number of hospitalizations due to exacerbations in the previous year increased the risk of readmission by 4.44 times [27]. The results of these studies supported the findings in this study, showing that patients with acute exacerbations within the previous 1 year had a 4.086-fold higher risk of readmission than those without acute exacerbations within the previous 1 year. The CAT score is a questionnaire as a simple, and quick instrument for measuring the severity and impact of symptoms in COPD patients and determining of the appropriate treatment for those patients in clinical practice [28]. Multiple studies have revealed that CAT score had good internal consistency and test–retest reliability for both stable and exacerbating COPD [29]. The CAT scores were higher in patients with a history of frequent exacerbations [30]. Herein, patients with higher CAT scores were associated with a higher risk of readmission within one year. This maybe because higher CAT scores were correlated with higher concentrations of serum C-reactive protein and plasma fibrinogen, demonstrating that systemic inflammation were more serious in patients with higher CAT scores [31]. For AECOPD patients with high CAT score, timely intervention should be provided and after discharge, follow-up should be conducted regularly to prevent the occurrence of readmission. At present, the aim of the treatment for COPD patients was to prevent the deterioration of lung function and alleviate symptoms to decrease the risk of exacerbations [32] and short-acting bronchodilators and long-acting bronchodilators including LAMA, LABA, SAMA, SABA and ICS are frequently applied in the treatment of COPD [33]. SABA is often applied on an as-needed basis for symptom relief in COPD patients [34]. However, previous studies also identified that the application of SABA might have a higher risk of readmission for than other therapies including arformoterol tartrate [35, 36]. In our study, SABA was identified as an important variable influencing the risk of readmission in AECOPD patients within one year after discharge. ICS application reduce the frequency and severity of exacerbations [37]. In the present study, patients receiving ICS treatment decreased the risk of readmission than patients without ICS treatment, which was supported by previous studies. For patients with AECOPD, ICS treatment should be appropriately provided to prevent the occurrence of readmission of those patients [38]. Another study also delineated that the incidence of adverse events was higher in patients with LABA treatment (50.2%) than patients without LABA treatments and exacerbation of COPD was the most commonly reported adverse events [39]. This may result in the increase of readmission of patients, which gave support to the results of our study, depicting that LABA treatment may cause a higher risk of readmission in patients [34, 35]. For AECOPD patients, LABA usage should be applied with caution.

This study measured the predictors for readmission in patients with AECOPD within one year and established two prediction models including logistic model and XGBoost model. Random forest filling method was used for dealing with the missing values via constructing multiple decision trees, and the data after filling have randomness and uncertainty, which can better reflect the real distribution of these unknown data. Random forest filling method can be well applied to high-dimensional data filling because each branch node selects random partial features instead of all features in the process of decision tree construction. High accuracy and reliability of the data after filling were ensured. The validation of the predictive values of the models were performed in the testing set. Due to the small sample size in our study, the training set included 70% of the samples and the testing set included 30% of the participants. This split ensured enough samples for construction of more reliable models and meanwhile, there were still some samples to validate the performance of the model. The ROC curves were drawn to display the results of respective models. The AUC values of the logistic model were good in the training set but poor in the testing set while the XGBoost model showed good predictive abilities in both the training set and the testing set. This indicated that XGBoost model was better than logistic model in predicting the risk of readmission in patients with AECOPD within one year. Currently, the prediction of the risk of readmission in patients with AECOPD was focused on 30 days and 90 days after treatment and discharge [10–12, 40], but 30 days or 90 days could not actually represent the disease procession. The readmission of AECOPD patients within one year indicated the long-term prognosis of patients. A previous study established a prediction model for one-year readmission of COPD patients, but the AUC value was only 0.703 and the results were not validated [16]. Compared with the former prediction models, we compared logistic model and XGBoost model and found the AUC values of the XGBoost model were higher in both the training set and the testing set. The variables involved in our model were common for clinicians to collect, and based on these variables, XGBoost model can quickly predict the possibility of readmission in AECOPD patients after discharge within one year. In addition, our prediction model was uploaded to the GitHub with a free access for everyone (https://github.com/shipingchenmedicine123/XGBoost-model). The instructions for using the model was shown in Additional file 1: File 1. We welcome more clinicians to use our model to validate the results of our study. The findings of the current study might help early identify patients with a high risk of relapse and readmission, especially patients with moderate exacerbations, and provide timely intervention measures to reduce the incidence of AECOPD and readmission to improve the prognosis of COPD patients and delay the progression of the disease.

The strengths of this study were that we dealt with the missing data and no bias were obtained, and the results might be more reliable. Internal validation was also conducted to verify the results of the present study. There were several limitations in our study. Firstly, the sample size was small and collected from a single center, which might decrease the statistical power especially in some variables with limited samples, such as patients SABA usage. Therefore, results of our study might be interpreted with caution. Secondly, external validation of the findings was not performed. Thirdly, subgroup analysis was not performed on patients with mild exacerbations, moderate exacerbations and severe exacerbations. In the future, well-designed studies with large scale of sample size from muti-centers and external validations were required to verify the results of the present study.

## Conclusions

In the current study, we constructed two models to predict the risk of readmission within one year in the AECOPD patients based on the predictors including acute exacerbation within the previous 1 year, LABA, ICS application, ALT level and the total CAT score. The results showed that the XGBoost model showed better predictive value in predicting the risk of readmission within one year in AECOPD patients than the logistic model. Variables including acute exacerbation in previous 1 year, the CAT score, and SABA and LABA application were more important in the XGBoost prediction model. The findings of our study might help identify patients with a high risk of readmission in patients with AECOPD within one year and provide timely interventions and treatment to prevent the reoccurrence of AECOPD.

## Supplementary Information

**Additional file 1:** Instuctions for using the XGBoost.

## Data Availability

The datasets used and/or analyzed during the current study can be made available from the corresponding author on reasonable request.

## Abbreviations

COPD: Chronic obstructive pulmonary disease
AECOPD: Acute exacerbation of COPD
BMI: Body mass index
LAMA: Long-acting muscarinic antagonist
LABA: Long-acting β agonist
SAMA: Short-acting muscarinic antagonist
SABA: Short-acting β agonist
PDE4I: Phosphodiesterase 4 inhibitor
ICS: Inhaled corticosteroids
Hb: Hemoglobin
RBC: Red blood cells
WBC: White blood cells
PLT: Platelets
NEUT: Neutrophil count
LYM: Lymphocyte count
MONO: Monocytes count
EOS: Eosinophil count
RDW: Red blood cell distribution width
ALT: Glutamic-pyruvic transaminase
AST: Glutamic oxalacetic transaminase
TBIL: Total bilirubin
ALB: Albumin
BUN: Blood urea nitrogen
Cr: Creatinine
mMRC: Modified Medical Research Council
CAT: COPD assessment test
FEV1: Forced expiratory volume in 1 s
LVEF: Left ventricular ejection fraction
