# Supplementary material for: Prediction of readmission in patients with acute exacerbation of chronic obstructive pulmonary disease within one year after treatment and discharge
Source: BMC Pulm Med. 2021 Oct 15;21:320. doi: 10.1186/s12890-021-01692-3 (PMC8518323; doi:10.1186/s12890-021-01692-3)
Supplement: Supplementary file 1 — Additional file 1: Instuctions for using the XGBoost. [file 12890_2021_1692_MOESM1_ESM.docx]

Instructions for using XGBoost-model (XGBoost模型使用说明)

1. Save the following files (保存下列文件)

* XGBoost model file: gv_model.pkl

* Test file: x_test.csv

Instructions for running the code (代码运行说明)

In python 3, run the following code (在python 3 环境下，运行下列代码)

* import pandas (载入pandas包)

* import joblib (载入joblib包)

* gv_test=joblib.load("gv_model.pkl" # [Import the model files (载入模型文件)]

* x_test.pd.read_csv('x_test.csv') # [Import the test files (载入测试文件)]

* gv_test.predict_proba (x_test)[:,1] #[Obtain the predictive probability (得到预测值)]
